# Experiment and Analysis of Termination Robustness Design for 1200 V 4H-SiC MOSFET

**DOI:** 10.3390/nano15110805

**Published:** 2025-05-27

**Authors:** Mengyuan Yu, Yi Shen, Hongping Ma, Qingchun Zhang

**Affiliations:** 1 Institute of Wide Bandgap Semiconductors and Future Lighting, Academy for Engineering & Technology, Fudan University, Shanghai 200433, China; 2 Shanghai Research Center for Silicon Carbide Power Devices Engineering & Technology, Fudan University, Shanghai 200433, China; 3 Institute of Wide Bandgap Semiconductor Materials and Devices, Research Institute of Fudan University in Ningbo, Ningbo 315327, China

**Keywords:** SiC MOSFET, termination, breakdown voltage

## Abstract

This study investigates the degradation mechanisms of 1200 V SiC MOSFETs during High-temperature Reverse Bias (HTRB) reliability testing, focusing on breakdown voltage (BV) reduction. Experimental results reveal that trapped charges at the SiC/SiO_2_ interface in the termination region alter electric field distribution, leading to premature breakdown. To address this issue, an optimized termination structure is proposed, incorporating reduced spacing between adjacent field rings and additional outer rings. TCAD simulations and experimental validation demonstrate that the improved design stabilizes BV within 2% deviation during 1000 h HTRB testing, which significantly enhances termination robustness.

## 1. Introduction

Silicon carbide (SiC) metal–oxide–semiconductor field-effect transistor (MOSFET) technology is gaining much attention due to advantages such as low conduction loss, high blocking voltage, and high operating temperature [1,2,3,4,5,6]. Now, it is widely used in automotive vehicles, solar systems, auxiliary power, etc. Specifically, in electric vehicle application, SiC MOSFETs are taking the place of Insulated-gate Bipolar Transistors (IGBTs) in motor drive systems, since they can increase vehicle mileage and reduce battery capacity [7,8,9,10]. Thus, more and more Original Equipment Manufacturers (OEMs) are adopting SiC technology in their vehicles. With consumers’ demands for increased driving range and reduced battery charging time, the battery voltage is changing from 400 V to 800 V, which requires stronger capability to handle high voltages for power devices.

The termination area is critical part of SiC MOSFETs, guaranteeing high-voltage operation. For termination design, various solutions have been proposed in the previous literature, including floating field ring (FFR), junction termination extension (JTE) and hybrid structures [11,12,13,14,15,16,17,18,19,20,21,22,23]. The most commonly used termination techniques for 1200 V devices are FFR and JTE structures. The FFR structure can be formed simultaneously with the active cell structure, which is achieved by the same implantation as Pwell or P+; it does not need an extra mask, so it can reduce processing cost. However, the BV of the FFR structure is sensitive to the space between adjacent rings, and the ranges of BV within different wafers and lots depend on processing consistency. Meanwhile, for the JTE structure, an extra mask is required during processing. It is not sensitive to the line spacer, but it is sensitive to the implant dose, which is also a challenge for processing. Currently, both FFR and JTE termination techniques are used in commercial products. For electric vehicle application, device reliability is gaining more attention. SiC MOSFET devices should be robust enough to guarantee reliability in long-term operation [24,25,26,27,28,29]. For automotive application, the ability to pass AECQ-101 for discrete devices, and AQG-324 for power modules, is mandatory.

This article reports the investigation of SiC MOSFET reliability through HTRB testing. The underlying failure mechanisms are discussed and analyzed. The results reveal that device termination structure degradation is the root cause of failure, where trapped charges during HTRB testing alter the electric field distribution. To solve this issue, this article proposes an optimized termination design that demonstrates enhanced robustness against charge trapping effects, which can maintain stable drain-source leakage current (I_dss_) and breakdown voltage (BV). Both TCAD simulation and experimental validation confirm the effectiveness of the optimized design.

## 2. Experimental Details

### 2.1. Device Under Testing

**Figure 1:**
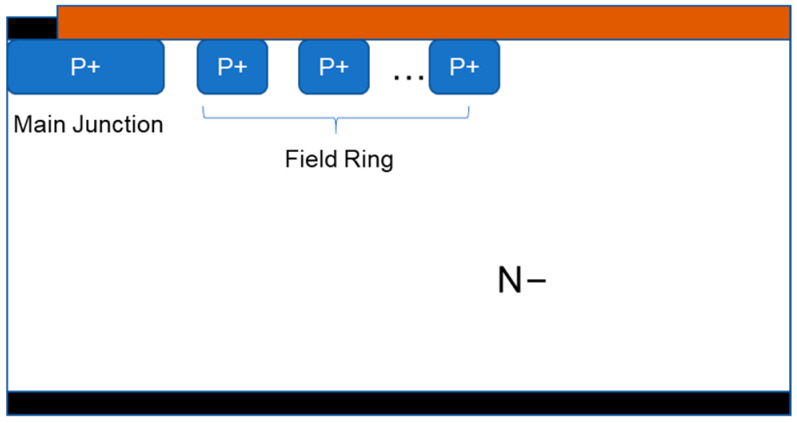
Field ring structure.

The devices used in the experiment were manufactured with the planar SiC MOSFET process, based on an n-type 4H-SiC epitaxy. The thickness and doping concentration of epitaxy were 11.5 μm and 9.5 × 10^15^ cm^−3^, respectively. Pwell, N+, and P+ regions were formed with high doses of aluminum and nitrogen implantation. In this work, the FFR structure was designed for the termination area because it is easily processed, and it is formed with P+ aluminum implantation. The structure of the termination is shown in Figure 1.

After device manufacture was finished, a chip probe (CP) test was carried out to check the electrical parameters, the result of which is shown in Table 1.

### 2.2. Experimental Conditions and Setup

For automotive application, AECQ-101 is a mandatory standard for power devices. In AECQ-101, HTRB/HTGB/H3TRB tests are the most critical, since they directly assess chip electrical reliability. For the HTRB test discussed in the article, the test conditions are listed in Table 2. The test voltage was performed at 100% nominal breakdown voltage, so, for the 1200 V SiC MOSFET device, the drain voltage was set to 1200 V. Gate voltage was set to 0 V to make sure the device channel was closed. The test temperature was set to the maximum operating temperature, which, in automotive application, is 175 °C. For the test lots and test numbers, AECQ-101 requires 3 different lots, and each lot needs at least 77 pcs samples. The test duration was 1000 h.

**Figure 2:**
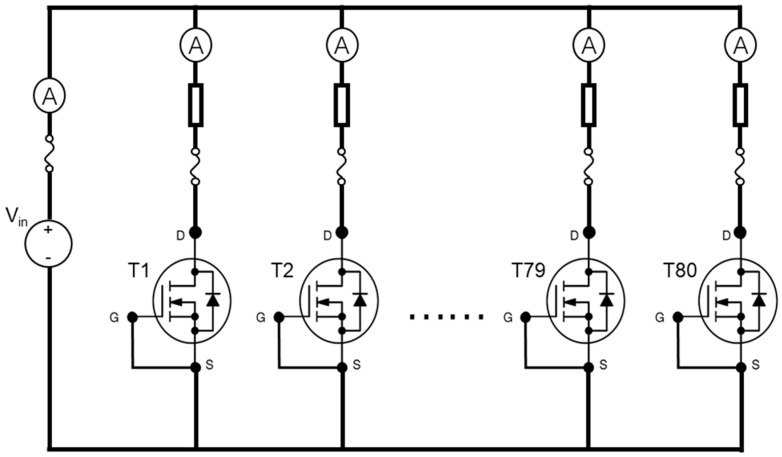
HTRB experimental setup.

The setup of the HTRB experiment is shown in Figure 2. G, D, and S are the abbreviations for gate, drain, and source, respectively. V_in_ is the input voltage source. There were 80 pcs SiC MOSFET devices parallel connected, and they were connected to the voltage source. The gate and source of the MOSFETs were short-circuited to make sure the channel is closed. For each branch, fuses and resistors were used to prevent overshoot current and protect the circuit if the device failed during the test. Furthermore, an ammeter was used to monitor the device leakage current.

## 3. Results and Discussion

**Figure 3:**
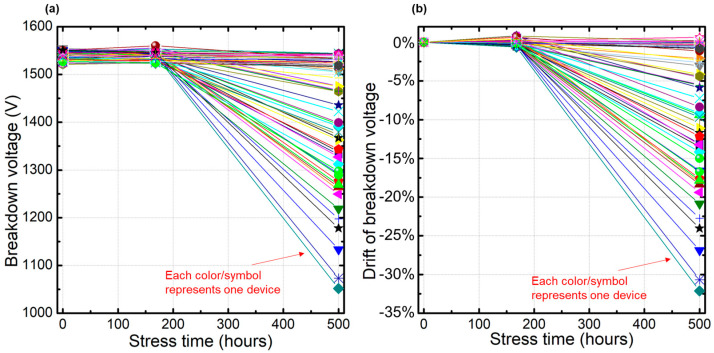
BV drops after HTRB test. (**a**) BV value after HTRB test (**b**) BV Drift after HTRB test.

The 80 pcs SiC MOSFET devices were randomly selected for the HTRB test. During the test, device parameters were tested at 0 h, 168 h, 500 h, and 1000 h. The gate-source leakage current, drain-source leakage current, breakdown voltage, threshold voltage, and conduction resistance were measured by Keysight B1506A under room temperature to check whether there was parameter shift after the test. Based on the test data, the changes in breakdown voltages and the drift compared to the initial values are plotted in Figure 3. As shown in Figure 3a, there are total 80 curves, each of which represents one device’s BV value change at 0 h, 168 h, and 500 h, while Figure 3b shows the drift in BV for the 80 pcs devices at 0 h, 168 h, and 500 h. The drift in breakdown voltage is defined as follows:

$$Drift\;of\;breakdown\;voltage=\frac{B{\left.V\right|}_{test\;hours}-B{\left.V\right|}_{0\;h}}{B{\left.V\right|}_{0\;h}}$$

In this HTRB test, after 500 h of testing, we found that the BV drops for most of the SiC MOSFET devices. As shown in Figure 3a, after 500 h, the BV of the 80 pcs devices declined in varying degrees. Their values dropped from the initial 1500 V to around 1000 V, while the maximum drift proportion after 500 h is more than 30%, which is shown in Figure 3b. The AECQ-101 failure criterion requires that the drift in breakdown voltage does not exceed 20% after testing. Obviously, this test can be judged as a failure based on this criterion, so the experiment ended prematurely after 500 h.

### 3.1. Device Failure Analysis

**Figure 4:**
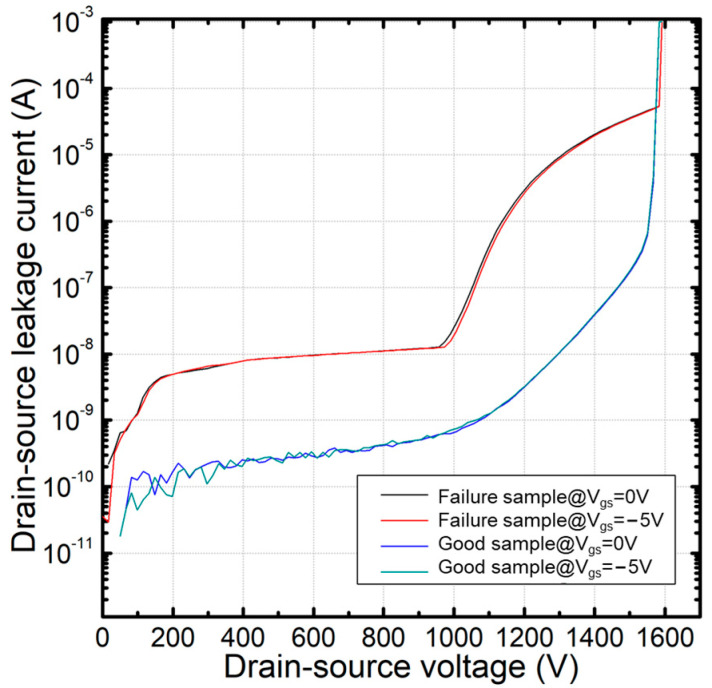
Comparison of BV curves between a good sample and a failure sample.

To determine the root cause of failure in BV drops, we selected several failure devices for analysis. The BV curves of a good sample and a failure sample are compared in Figure 4.

From the figure, we can obtain the following information:

1. The overall I_dss_ leakage current of the failure sample is higher than that of the good sample;
2. There is a sudden leakage current increase around 1000 V for the failure sample;
3. The BV curves remain the same, regardless of whether the gate voltage is 0 V or −5 V.

Combining the information above, we can ensure there is degradation for these devices. Furthermore, from point 3, we can conclude that the leakage current is not from the active cell area, but from the termination area. There is degradation in the termination area, and breakdown occurs when the drain-to-source voltage reaches 1000 V.

**Figure 5:**
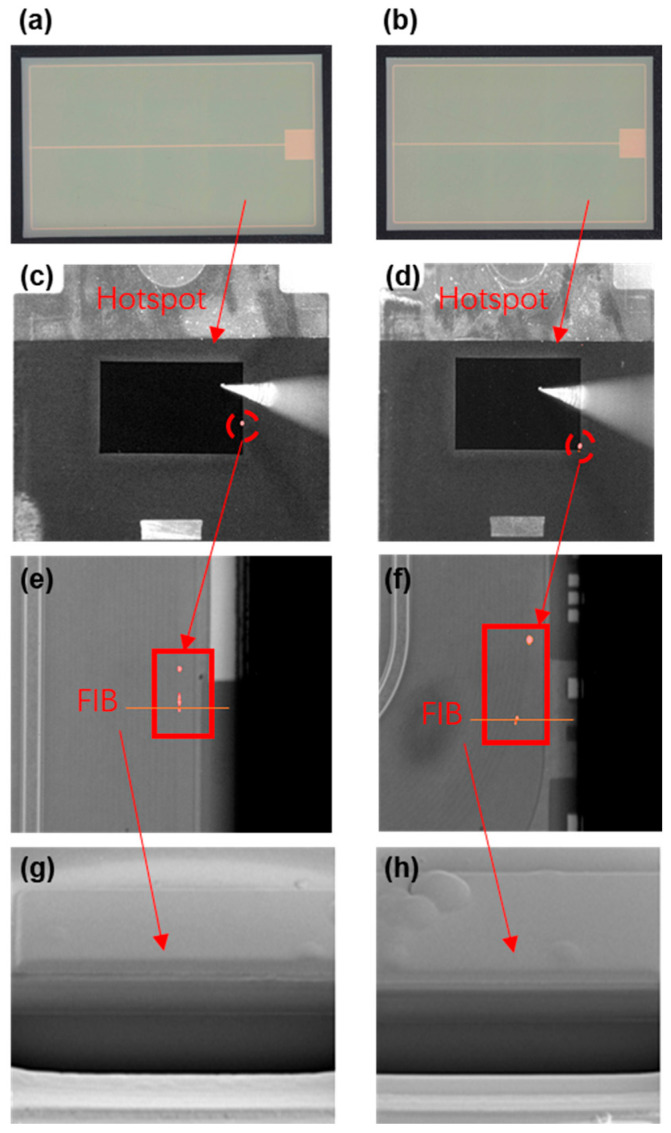
Hotspot and FIB analysis for two failure samples. (**a**,**b**) OM pictures from backside of chips (**c**,**d**) Hopspot analysis results (**e**,**f**) Enlarged view of hotspot area (**g**,**h**) FIB results.

To confirm the failure position, two failure samples were decapped, and hotspot analyses were performed on them. Figure 5a,b show the Optical Microscope (OM) pictures, which were observed from backside of the chips. No abnormalities were found from the OM results. However, the following hotspot analysis, shown in Figure 5c,d, implies that there is leakage current on the edge of the chip where the termination area is located. This result confirms our speculation: the I_dss_ leakage current increase and BV drop occurred because of termination degradation. By enlarging the hotspot area, we can further find that the leakage current is concentrated on the outer termination ring. Focused Ion beam (FIB) analysis was then implemented on the hotspot (the orange line in Figure 5e,f to see whether there were abnormal structures or particles that could lead to increased leakage current. The results are shown in Figure 5g,h, which indicate that there are no abnormal structures or defects inside the hotspot.

**Figure 6:**
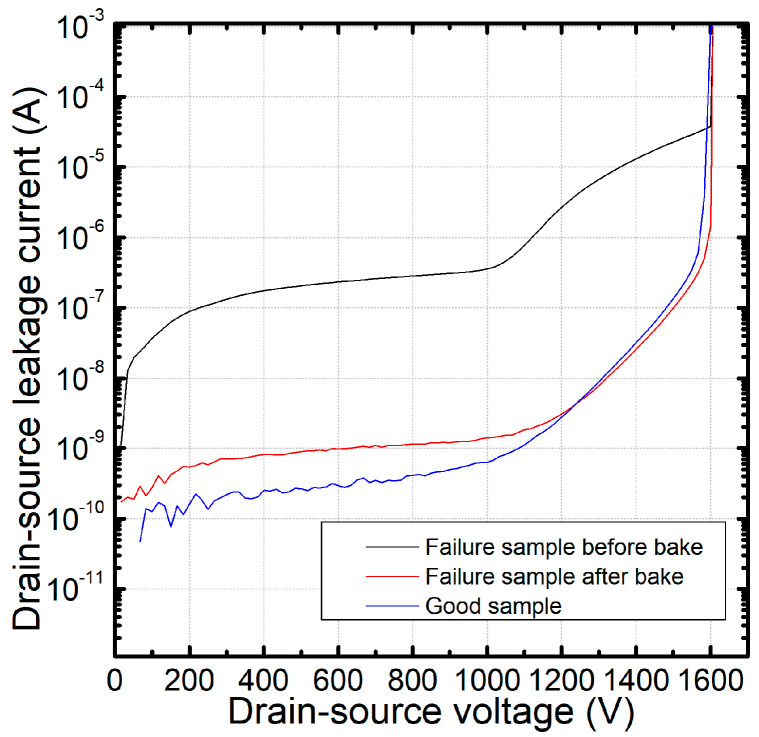
BV curve comparison before and after baking.

To further investigate the failure mechanism, the failure samples were baked under high temperatures. Their BV curves were tested before and after baking. The curves were also simultaneously compared with those of the good sample, the result of which are shown in Figure 6. We can find, for the failure samples before baking, that the I_dss_ current is higher than that of the good sample, which is consistent with Figure 4. However, after 2 h of baking at 175 °C, the failure sample BV curves were recovered, as the I_dss_ leakage decreased and their curves were almost the same as those of the good sample.

### 3.2. Failure Mechanism Discussion

**Figure 7:**
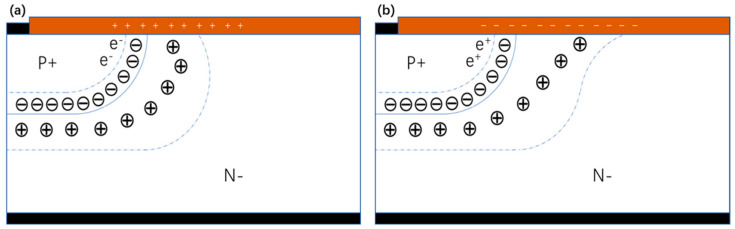
Influence of trapped charges to termination: (**a**) positive charge and (**b**) negative charge.

Combining the analysis information above, we can conclude that the root cause for failure in the leakage current increase and BV drop after the HTRB test is trapped charges in the SiC/SiO_2_ interface. The trapped charges in the interface influence the electric potential and space charge area, thus changing the electric field distribution and breakdown characteristics. As shown in Figure 7, if there are positive charges in the interface layer, negative charges are induced in the main junction, and then the electric field is crowed at the main junction, which leads to early breakdown of the main junction. Meanwhile, if there are negative charges in the interface layer, positive charges are induced in the main junction, which alleviates the electric field at the main junction. However, it will increase the electric field of the termination edge area, which could also lead to device leakage, current increase, and device characteristic degradation. Combining the failure analysis results, we can conclude that, during this HTRB test, there were negative charges trapped in the SiC/SiO_2_ interface of the termination area, which increased the termination leakage current and, thus, led to BV drops. After high-temperature baking, the charged traps were recovered, then the electric field distribution returned to the original design. Thus, the leakage current and BV curves recovered, as well.

**Figure 8:**
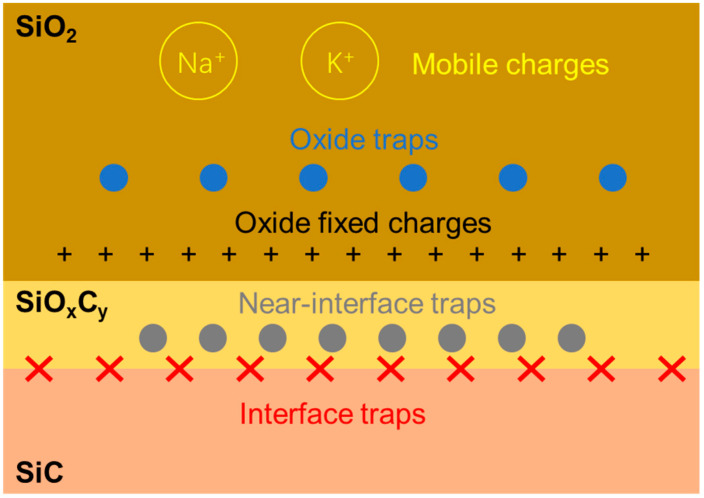
Trap distribution in and near the SiC/SiO_2_ interface.

The defects in and near the SiC/SiO_2_ interface, and their impacts on SiC MOSFET performance, have been reported in the literature [30,31,32,33,34]. Based on the distribution locations, they are classified as interface traps, near-interface traps, oxide traps, oxide fixed charges, and mobile charges. They are shown in Figure 8 below. The interface traps, located in the SiC/SiO_2_ interface, are caused by carbon cluster, carbon dangling bonds formed during the oxidation process. Near-interface traps, which exist in the transition layer SiO_x_C_y_, between SiC and SiO_2_, are primarily associated with oxygen vacancy defects in the oxide. Near-interface traps close to the interface can capture carriers through direct tunneling or interface state-assisted tunneling.

Interface traps and near-interface traps have serious impacts on device performance, such as low channel mobility, threshold voltage instability, poor oxide reliability, and unreliable termination stability, which is discussed in this article. They affect the transportation of electrons by capturing them, thus reducing the number of movable electrons in the channel and resulting in a degradation of channel mobility. With the long-term operation of the device, under a high electric field, the carriers can enter the oxide layer through the tunneling effect with the help of near-interface traps, which leads to threshold voltage shift. The accumulated trapped charges increase local field strength. They ultimately form conductive paths in the oxide layer, leading to reliability failure. This is precisely the issue encountered in the experiment described in this article.

## 4. TCAD Simulation and Optimized Design

**Figure 9:**
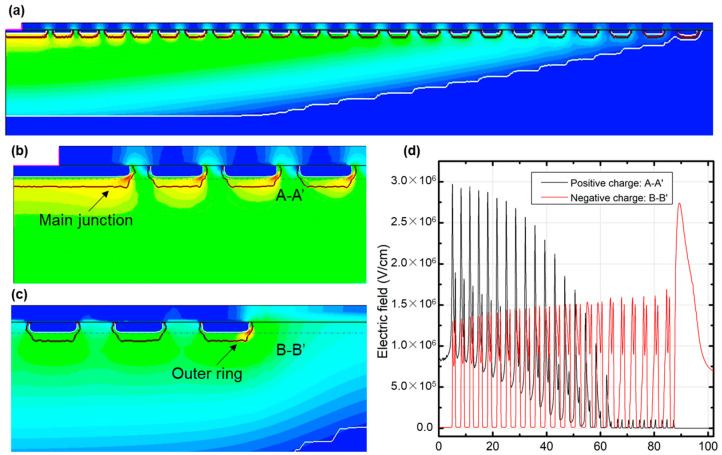
Electric field distribution under positive and negative trapped charges. (**a**) Termination structure (**b**) Enlarged view of main junction (**c**) Enlarged view of outer ring (**d**) Electric field distribution under both positive and negative charges.

TCAD simulation was conducted by Sentaurus to verify the failure mechanism. To simulate the sensitivity of the termination structure to trapped charges, the fixed charge in the interface of SiC/SiO_2_ was set in the range of −3 × 10^12^ cm^−2^ to 1 × 10^12^ cm^−2^. If the trapped charge was zero, the simulated BV was around 1500 V. However, if there was a trapped charge, the BV value dropped. In particular, when the trapped charge was −3 × 10^12^ cm^−2^, the BV value decreased the most, to only half of the original value. The electric field distribution under different trapped charges was analyzed. The termination structure is shown in Figure 9a, while Figure 9b,c are enlarged views of the main junction and the outer ring, respectively. If there was a positive charge, the maximum electric field was located at the main junction, as shown in Figure 9b. Meanwhile, if there was a negative charge, the maximum electric field was located at the edge of the termination, as shown in Figure 9c. By cutting the termination structure along the A-A’ and B-B’ lines, we obtained the electric field distributions under both positive and negative charges in Figure 9d. Trapped charges had significant impacts on the electric field distribution.

**Figure 10:**
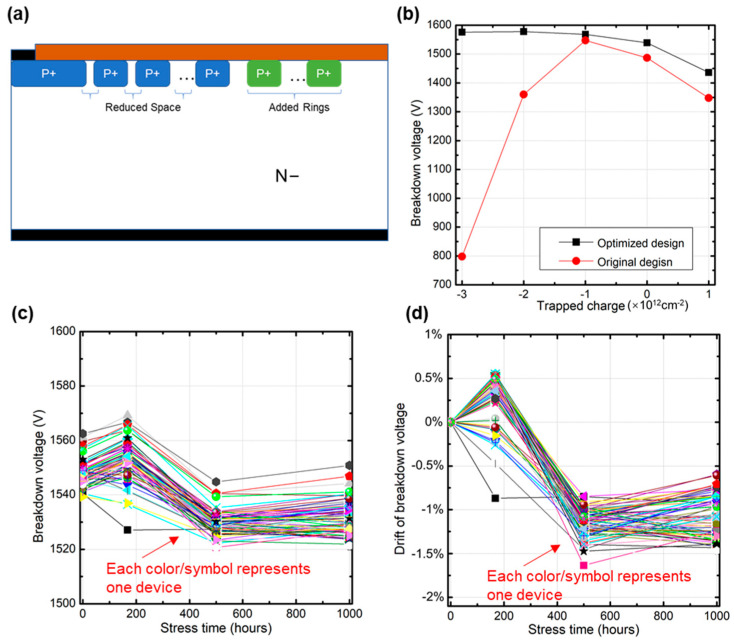
BV remained stable during HTRB testing for the optimized design. (**a**) Optimized termination design (**b**) BV comparison of optimized design and original design (**c**) BV value of optimized design after HTRB test (**d**) BV Drift of optimized design after HTRB test.

The termination structure needs to be optimized to improve its BV under both negative and positive trapped charges. Considering the electric field distribution shown in Figure 9, the electric field of both the main junction and the termination edge should be optimized. To alleviate the electric field strength at the main junction under a positive trapped charge, the space between adjacent rings should be reduced to provide better electric field shielding protection, while more rings should be added outside of the original structure to alleviate the electric field strength at the termination edge under negative trapped charges. The optimized termination structure is shown in Figure 10a. Its BV was compared with the original design under different trapped charges with TCAD simulation. The result in Figure 10b shows that the BV of the optimized design improved under both negative and positive trapped charges. It can be stable at around 1500 V under a wide range of trapped charges; thus, its robustness has been greatly improved.

New wafers were taped out based on their optimized termination structure. The chips were packaged in TO-247 devices, and then 80 pcs devices were randomly selected to be qualified with HTRB testing again. Device parameters were tested at 0 h, 168 h, 500 h, and 1000 h. Test results are shown in Figure 10c,d. Both of them contain 80 curves. Each of the curves represents one device’s BV value change and its corresponding drift. We can see that, after the 1000 h HTRB test, the BV remained stable compared with the original values. The drift of BV before and after HTRB testing was less than 2%, which verifies the robustness of the optimized termination structure.

## 5. Conclusions

The BV of SiC MOSFETs dropped during HTRB testing. Failure analysis results showed that the leakage current of the termination area increases, which is caused by trapped charges. To solve the problem, an optimized termination robustness design for 1200 V SiC MOSFET is proposed in this article. By reducing the space between adjacent rings and adding more rings outside of the original termination structure, both the electric field strength of the main junction and the termination edge were alleviated. TCAD simulation showed that its robustness was greatly improved under a wide range of trapped charges. New chips were manufactured with the optimized termination structure and qualified with HTRB testing again. Results showed that the BV was stable after HTRB testing, and the drift was less than 2%, validating that the robustness was greatly improved with the optimized termination design.

## Figures and Tables

**Table 1:** Chip probe electrical parameters.

Item	Test Condition	Value
I_gss_	V_gs_ = 25 V, V_ds_ = 0 V	4 nA
I_dss_	V_ds_ = 1400 V, V_gs_ = 0 V	17 nA
Vth	V_gs_ = V_ds_, I_d_ = 20 mA	2.8 V
R_dson_	V_gs_ = 18 V, I_d_ = 60 A	17 mΩ
BV_DSS_	V_gs_ = 0 V, I_d_ = 5 uA	1540 V

**Table 2:** HTRB test conditions.

Main Parameters	Value
Drain voltage	1200 V
Gate voltage	0 V
Test temperature	175 °C
Test number	≥77 pcs
Test lot	3 lots
Test duration	1000 h

## Data Availability

The data presented in this study are available on request from the corresponding authors.
